# Cellular and humoral immune responses in cats vaccinated with feline herpesvirus 1 modified live virus vaccine

**DOI:** 10.3389/fvets.2024.1516850

**Published:** 2025-01-15

**Authors:** Hongchao Wu, Peipei Qiao, Yunyu Chen, Caihong Liu, Ningning Huo, Hangtian Ding, Xiaojuan Wang, Lulu Wang, Xiangfeng Xi, Yuxiu Liu, Kegong Tian

**Affiliations:** ^1^College of Veterinary Medicine, Henan Agricultural University, Zhengzhou, China; ^2^National Research Center for Veterinary Medicine, Luoyang, China

**Keywords:** feline herpesvirus 1, MLV vaccine, cats, cellular immune responses, IFNγ

## Abstract

Feline herpesvirus 1 (FHV-1) is an important pathogen causing infectious rhinotracheitis in felids, mainly infecting the upper respiratory tract and conjunctiva. Multiple vaccines are available to prevent FHV-1 infection, and the antibody levels are always used to evaluate their effectiveness. However, the cellular immunity response following immunization in cats remains unclear. This study investigated the immune responses (humoral and cellular) in cats immunized with the FHV-1 modified live virus vaccine. The results indicated that vaccination significantly reduced clinical signs, and antibody levels, including virus-neutralizing (VN) antibodies and immunoglobulin G (IgG), in the vaccine group were higher than those in the control groups. Additionally, the vaccine significantly increased cytokine secretion, indicating Th1-type cellular immune responses in cats. Moreover, cellular immune-related indicators, such as CD8+ T cells, CD4+ T cells, and interferon-gamma levels, were inversely correlated with clinical signs post-challenge by FHV-1 in vaccinated cats, highlighting its crucial role in protecting cats against FHV-1 infection. In summary, this study demonstrated the importance of cellular immune responses in protecting cats from FHV-1 infection after vaccination.

## 1 Introduction

Feline herpesvirus 1 (FHV-1), or felid herpesvirus 1 (FeHV-1), belongs to the Var*icellovirus* genus in the *Alphaherpesvirinae* subfamily of the *Herpesviridae* family (1). FHV-1 is highly contagious and an important pathogen, causing infectious rhinotracheitis in felids (2). The kittens are the most susceptible to infection, with morbidity and mortality rates up to 100 and 50%, respectively (3, 4). FHV-1 mainly infects the upper respiratory tract and conjunctiva, which clinically manifests fever, conjunctivitis, keratitis, nasal and ocular serous or mucinous discharge, and severe infection can cause lobular pneumonia and periocular skin ulcers (5, 6). Similar to other herpesviruses, the incubation period is established in the trigeminal ganglion following the acute phase of infection (7). When the cats are under stress or immunosuppression, the virus is easily reactivated, accompanied by the periodic shedding of infectious viruses and the recurrence of clinical symptoms, resulting in lifelong virus infection and recurrence (1). As part of the feline core vaccine, FHV-1 is included in a triple combination of inactivated and modified live virus (MLV) vaccines. The World Association of Small Animal Veterinarians (WSAVA) recommends vaccinating kittens with FHV-1 at 6–8 weeks of age, with at least one more dose after 2–4 weeks, followed by annual vaccination thereafter (8).

Despite widespread vaccination worldwide, current FHV-1 vaccines offer limited and short-duration protection. The diverse evasion strategies employed by herpesviruses to counteract the host’s innate immune response and their encoding of various anti-interferon response effectors often result in a limited efficacy of FHV-1 vaccination (9). FHV-1 comprises 11 distinct proteins that collaborate to facilitate immune evasion during infection (10). The subsequent viral infection outbreaks cannot be disregarded even though MLV vaccines induce more robust immune responses than inactivated vaccines (11, 12). Moreover, serum virus antibody levels are frequently applied to assess immunity to FHV-1; however, the impact of cellular immune response should not be overlooked on immunity against infections (1).

Vaccination induces a critical cellular immune response essential for preventing viral infections. Upon antigen exposure, the immune system initiates a robust response by activating T lymphocytes, specifically CD4+ helper and CD8+ cytotoxic T cells, which recognize and eliminate infected cells (13). Interferon-gamma (IFN-*γ*), a key cytokine secreted by activated T cells, plays a crucial role in this process. It binds to receptors on infected cells, triggering a cascade of intracellular events that effectively inhibit virus replication and dissemination (14). IFN-*γ* also enhances the function of other immune cells, coordinating a synchronized attack on the virus (15). This coordinated cellular immune response is essential for effective viral clearance and overall health maintenance (16). Observed that the activation of T cells to produce IFN-*γ* is the predominant immune response after a bovine herpesvirus 1 (BHV-1) infection. Furthermore, the bacterium-like particle (BLP) vaccine with FHV-1 gB, gC, and gD induces a specific cellular immune response in mice (17). However, the cellular immune responses induced in cats after FHV-1 infection or vaccination and the indicators associated with resistance to FHV-1 infection remain unclear.

This study focused on exploring immune responses in immunized cats with the FHV-1 MLV vaccine and subsequent challenges with FHV-1. Moreover, a correlation between humoral and cellular immune responses and protection against challenge was analyzed to obtain indicators related to protection against FHV-1 infection. Furthermore, our findings will serve as a reference for the assessment of vaccines related to FHV-1.

## 2 Materials and methods

### 2.1 Animals

A total of 13 eight-week-old cats free of feline calicivirus (FCV), feline parvovirus (FPV), FHV-1, and Feline coronavirus (FCoV) were randomly divided into three groups. Each cat was kept in a separate cage, with different groups separated into distinct spaces. The animal studies were approved by the Committee on the Ethics of Animal Experiments of the National Research Center for Veterinary Medicine (Permit Number: 202307001) and conducted in conformity with the Guide for the Care and Use of Animals in Research of the People’s Republic of China.

### 2.2 Cells, viruses, and vaccine preparation

Feline kidney cells (F81 cells) were maintained in Roswell Park Memorial Institute (RPMI) 1,640 medium (Gibco, Carlsbad, CA, United States), supplemented with 8% fetal bovine serum (Cegrogen Biotech, Germany), 100 U/mL penicillin, and 0.1 mg/mL streptomycin (Sigma-Aldrich, St. Louis, MO, USA) at 37°C with 5% CO_2_. The FHV-1 strain 64 was isolated from the eyelid and nasal swab suspension of FHV-1 infected cats in Henan Province in 2016. The eyelid and nasal swab suspension were inoculated on F81 cells for virus isolation as previously described (18). When CPE were observed in 80% of the cells, cultures were harvested and the resultant virus stocks were stored at −80°C and named as FHV-1 strain 64. Cats infected with FHV-1 strain 64 presented with ocular and nasal discharge, conjunctival congestion, and sneezing (data not shown). The serial passage was used to attenuate the FHV-1 strain 64, and the MLV vaccine was prepared by attenuating the FHV-1 strain 64 (10^6.0^ TCID_50_/mL).

### 2.3 Experimental design

**Figure 1:**
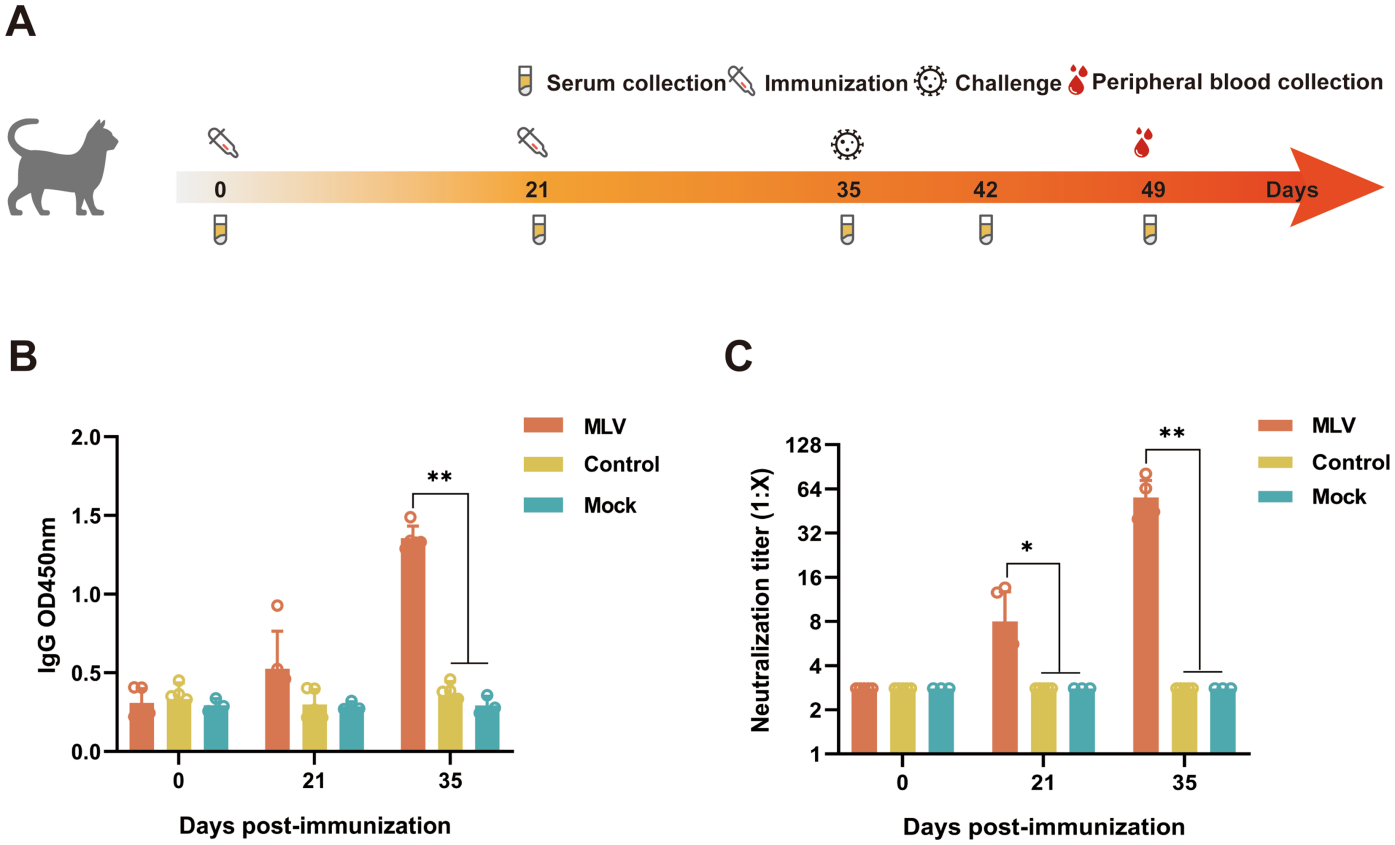
Detection of antibody levels in the serum from the immunized cats. **(A)** Schematic diagram of the experiment. **(B)** The serum IgG was measured using ELISA at OD450 nm (*n* = 13). **(C)** The serum FHV-1 neutralizing antibody titers were measured using FHV-1 neutralization assay (*n* = 13).

The experimental schedule is illustrated in Figure 1A. The cats were randomly divided into three groups: (1) Unvaccinated controls, *n* = 5, (2) vaccine immunization group, *n* = 5, (3) Mock group, *n* = 3; based on the immunization schedule of the vaccine in the published article, cats were immunized subcutaneously (SC) on days 0 and 21 (19, 20). Groups 1 and 2 were challenged with intranasal FHV-1 64 on day 35, and the mock group was inoculated with RPMI 1640 medium. Clinical symptoms and body temperatures were monitored daily, and venous blood was collected on days 0, 21, and 35 to detect the virus-neutralizing (VN) antibody and IgG levels. Serum cytokine levels were detected in venous blood collected on days 0, 21, 35, 42, and 49, and serum was stored at −20°C for further testing. On day 49, peripheral blood was collected to isolate lymphocytes, after which the cats were humanely euthanized.

### 2.4 Virus-neutralizing antibody detection

The serum was analyzed for FHV-1 VN antibodies by heat-inactivating it at 56°C for 30 min, followed by a two-fold serial dilution ranging from 1:2 to 1:256 according to the previous method (21). A 200 TCID_50_ dose of FHV-1 was mixed with the serum sample in a volume ratio 1:1, incubated at 37°C for 1 h, and then added F81 cells (2 × 10^4^ cells/100 μL). After a 4-day incubation at 37°C with 5% CO_2_, antibody titers were measured by CPE and expressed as the reciprocal of the highest dilution at which infection of the F81 cells was inhibited in 50% of the culture wells.

### 2.5 IgG antibody detection

The IgG antibodies in the serum were detected 14 days after the second immunization using an indirect enzyme-linked immunosorbent assay (ELISA) according to the previous method (17). Microtiter plates (96-well) were coated with lab-stored, purified FHV-1 (0.25 μg/well) and incubated overnight at 4°C. The plates were then blocked with a blocking buffer for 24 h at 4°C. Serum samples were heat-inactivated at 56°C for 30 min and pre-diluted (1:2,000) with phosphate buffer saline (PBS). The samples were then incubated at 37°C for 1 h, followed by incubation with HRP-conjugated goat anti-feline IgG (1,2000) at 37°C for 30 min. Tetramethyl benzidine (TMB) was added to each well, and then the reaction was stopped with 2 M H_2_SO_4_. The absorbance was measured at an optical density (OD) of 450 nm using a microplate reader (BioTek, Vermont, United States).

### 2.6 Clinical signs and histological analysis

Clinical scores were obtained by trained observers masked to the vaccine groups from day 0 to 14 days after the FHV-1 challenge (day 35), as described in previous studies (22, 23). Two observers recorded signs for 30 min per group each morning. Clinical signs included fever, conjunctivitis, blepharospasm, ocular discharge, sneezing, and nasal discharge. The eyelids, turbinates, tracheas, and lungs of cats were harvested 14 days after the FHV-1 challenge. All tissue samples were fixed in 4% paraformaldehyde, embedded in paraffin, and sectioned before hematoxylin and eosin staining (H&E). Monoclonal antibody 5H8 against FHV-1 gD and goat anti-mouse IgG conjugated to HRP (ThermoFisher Scientific, Waltham, MA, United States) were used for immunohistochemical (IHC) assays. Stained sections were visualized using a microscope (Leica DM2500, Leica Microsystems, Germany).

### 2.7 Viral shedding

After challenge, the eyelid swabs were tested daily for the FHV-1 DNA. Eyelid swabs were collected into 1 mL of PBS, and viral nucleic acid was extracted from 200 μL eyelid swab samples using the Viral Nucleic Acid Extraction Kit (Geneaid Biotech Ltd., Taiwan, P.R. China). The primers for the PCR test of FHV-1 as previously described (21). Briefly, the primers used were: Forward (5′-3′): GAC GTG GTG AAT TAT C; Reverse (5′–3′): CAA CTA GAT TTC CAC CAG GA. The reaction conditions were as follows: pre-denaturation at 95°C for 3 min, denaturation at 95°C for 30s, annealing at 58°C for 30s, elongation at 72°C for 30s, 30 cycles. Amplicons were detected on 1.5% agarose gels, with a positive reaction indicated by a 288 bp band.

### 2.8 Feline inflammatory cytokines detection

The feline interferon-gamma (IFN-*γ*) ELISA kit (Mabtech AB, Stockholm, Sweden), feline interleukin 2 (IL-2), and IL-4 ELISA kit (R&D Systems, Minneapolis, United States), and feline IL-17A ELISA kit (MyBioSource, San Diego, CA, United States) were used to measure the levels of IFN-γ, IL-2, IL-4, and IL-17A, respectively, following the manufacturer’s instructions. Each sample was analyzed in duplicate.

### 2.9 Flow cytometry

The peripheral blood lymphocytes were isolated with commercial kits (Tianjin Haoyang Biological Manufacture, P.R. China) following the manufacturer’s instructions and stored in liquid nitrogen. The percentages of CD4+ and CD8+ T cells in lymphocytes were determined by flow cytometry using a staining method adapted from a previously published protocol (24). The thawed lymphocytes were washed thrice with flow cytometry staining buffer by centrifugation at 2000 rpm for 5 min each. The lymphocytes were blocked by resuspending for 10 min at 4°C in flow cytometry staining buffer containing 10 μL negative cat serum. Subsequently, a total of 1 × 10^6^cells were stained with 10 μL FITC Anti-cat CD4, RPE Anti-cat CD8, and Alexa Fluor^®^ 647 Anti-cat CD134 antibodies (Bio-Rad Laboratories, United States) for 30 min at 4°C in the dark. After washing, the lymphocytes were stained with Hoechst 33258 (ThermoFisher Scientific, Waltham, MA, United States) for 20 min at 4°C in the dark. After three additional washes, the lymphocytes were resuspended with a flow cytometry staining buffer and analyzed using a flow cytometer (BD Biosciences, Franklin, CA, United States).

### 2.10 Enzyme-linked immunospot assay

The feline IFN-*γ* ELISpot kit (Mabtech AB, Stockholm, Sweden) and feline IL-2 ELISpot kit (R&D Systems, Minneapolis, United States) were used to evaluate the cell-mediated immune response induced by the FHV-1 vaccine. Lymphocytes from the cats on day 49 obtained as described previously were seeded into ELISpot plates (5 × 10^5^ live cells/well) and stimulated with FHV-1 (MOI = 0.3) for 24 h. The assay was performed according to the manufacturer’s instructions. The spot-forming cells (SFCs) were counted using an ELISpot plate reader (Cellular Technology Ltd., Shaker Heights, OH, United States). The IFN-*γ* or IL-2 secretory cell numbers were calculated by subtracting the values from PBS-stimulated wells from FHV-1 stimulated wells.

### 2.11 Statistical analysis

The total clinical scores for each cat were calculated over the post-challenge (days 0–14) study periods. A Statistical Package for the Social Sciences (version 21.0) was used to conduct Pearson correlation analysis between clinical scores post-FHV-1 challenges and detection indicators. Data were analyzed by one-way analysis of variance and an independent-sample *t*-test at a significance level of *p* < 0.05.

## 3 Results

### 3.1 FHV-1 MLV vaccine immunization induces efficient antibody production in cats

The immunogenicity induced by the FHV-1 modified live virus vaccine was evaluated by administering two injections, 21 days apart, to cats (Figure 1A). The results showed that the immunized cats produced significantly higher levels of IgG compared to the control group at 14 days after the second immunization (Figure 1B). Similarly, the neutralizing antibody levels were notably higher in the immunized cats than those of non-immunized cats (Figure 1C). These data demonstrate that the FHV-1 MLV vaccine effectively induced IgG and VN antibodies production against FHV-1 infection in cats.

### 3.2 Proportion of CD4+ and CD8 + T cells

**Figure 2:**
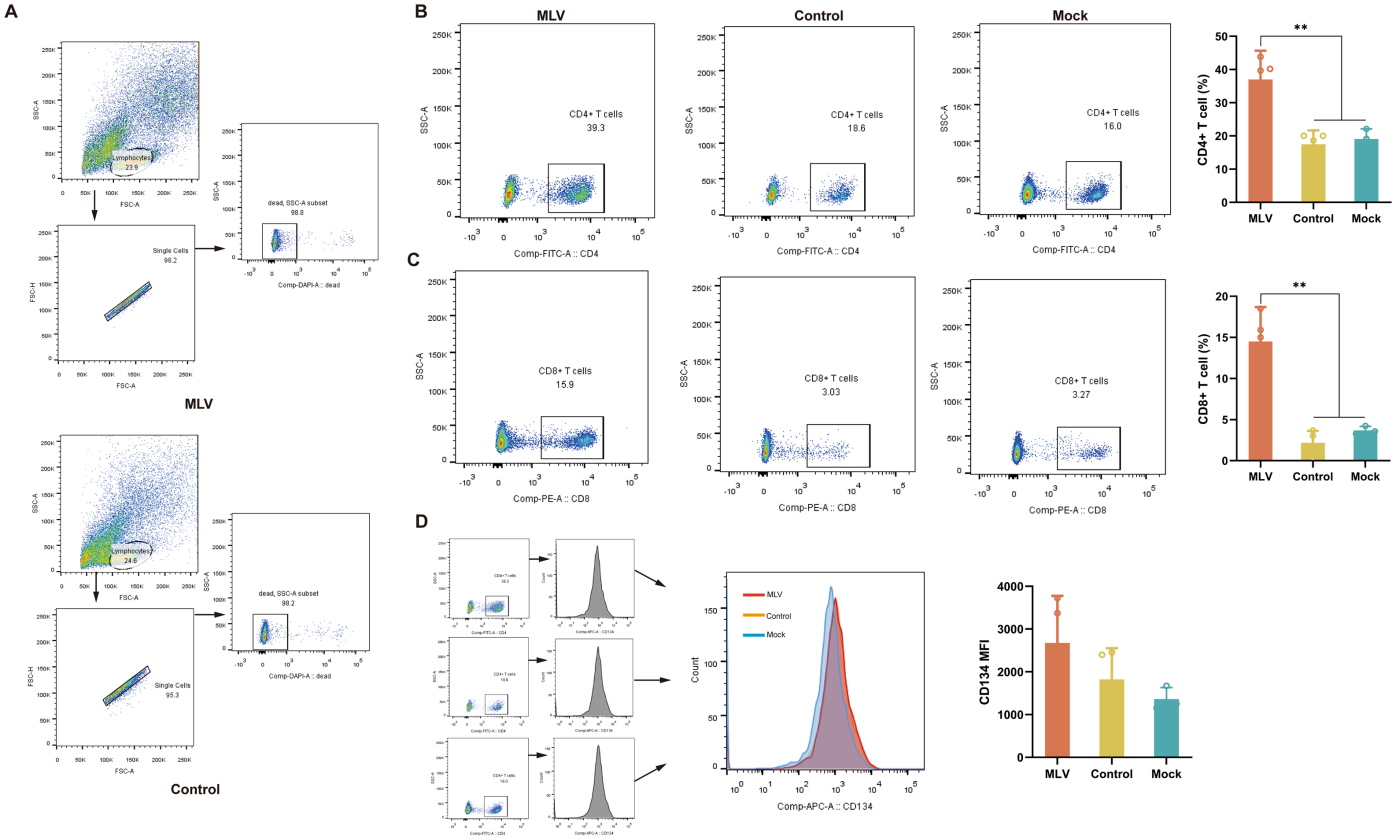
Analysis of T cells in lymphocytes. On the 14th day after the challenge, peripheral blood lymphocytes of cats were collected. **(A)** The gating strategy of CD4+ T cells and CD8+ T cells in flow cytometry. **(B)** Representative flow cytometric plots of lymphocytes and the percentage of CD4+ T cells (*n* = 11). **(C)** Representative flow cytometric plots of lymphocytes and the percentage of CD8+ T cells (*n* = 11). **(D)** Representative flow cytometric plots of lymphocytes and the MFI of CD4+ CD134+ T cells are representative of activated CD4+ T cells (*n* = 11). The numbers in the gates refer to the percentage of positive cells for each marker. The results were considered statistically significant at *p* < 0.05.

CD8+ and CD4+ T cells play a crucial role in clearing viral infections. To further investigate the T cell responses induced by the FHV-1 MLV vaccine, the percentages of CD8+ and CD4+ T cells within the lymphocytes of vaccinated cats were measured using flow cytometry. Figure 2A depicts the gating strategy of CD4+ T cells and CD8+ T cells in flow cytometry. The results revealed a substantial increase in the CD4+ T cells in the immunized cats compared to the control and mock group (Figure 2B; *p* < 0.05). The immunized group also exhibited a significantly increased number of CD8+ T cells than the control and mock group (Figure 2C; *p* < 0.05). Furthermore, the mean fluorescence intensity (MFI) of CD134+ T cells was also detected as a result of CD134 being expressed on activated CD4 T cells (25). However, there was no significant increase in the MFI of CD134+ T cells in the immunized group compared to the control and mock group (Figure 2D; *p* > 0.05).

### 3.3 FHV-1 MLV vaccine immunization drives Th1-based cellular immune responses in cats

**Figure 3:**
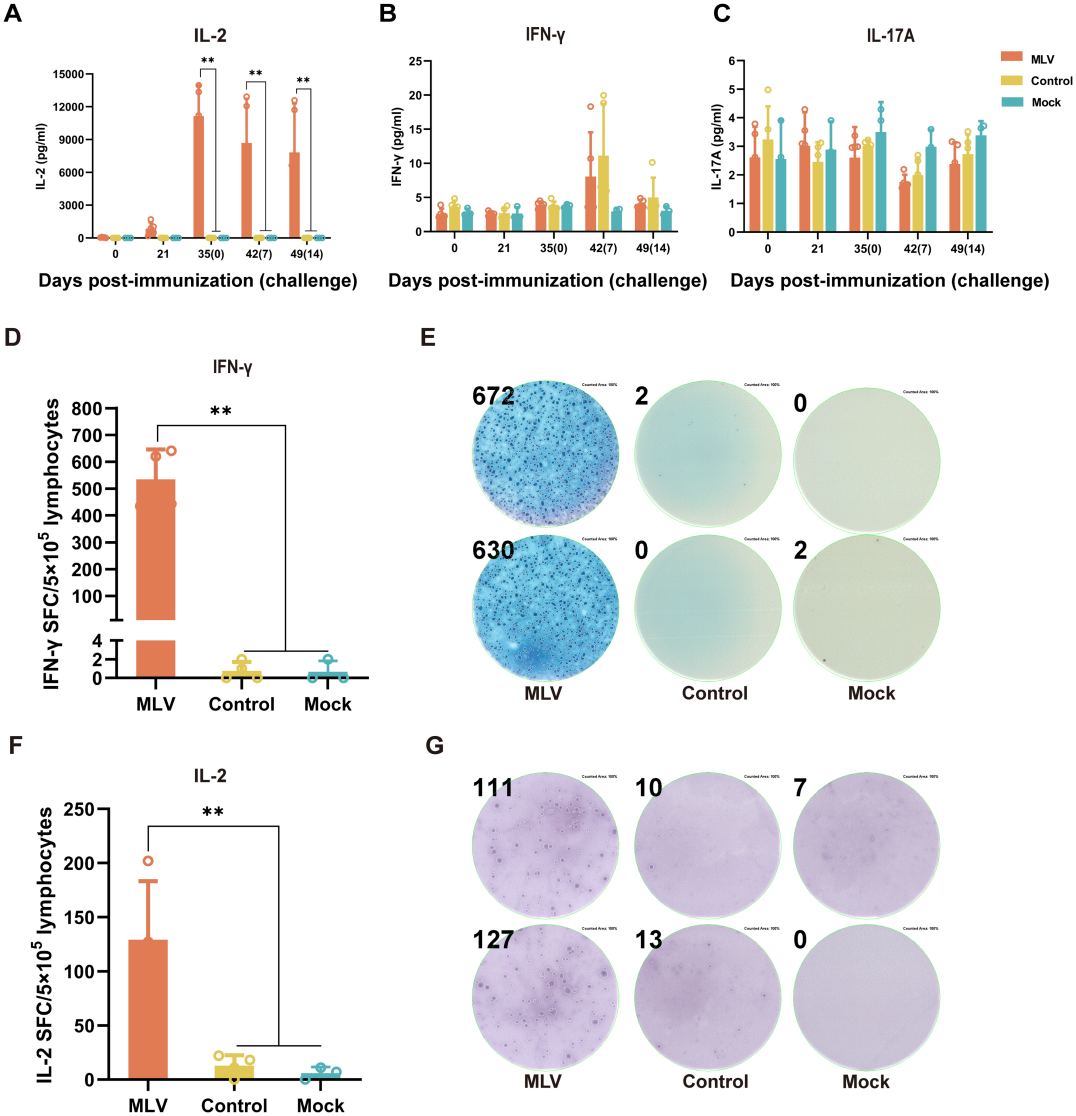
The FHV-1 MLV vaccine immunization induces Th1-based cellular immunity in cats. **(A–C)** The cytokine levels of IL-2, IFN-*γ*, and IL-17A in cat serum after immunization or challenge (*n* = 13). **(D)** Number of IFN-γ spot-forming counts after 24 h of stimulation with FHV-1 (*n* = 11). **(F)** Number of IL-2 spot-forming counts after 24 h of stimulation with FHV-1 (*n* = 11). **(E,G)** Representative images of ELISpot wells. The results were considered statistically significant at *p* < 0.05.

To assess the activation of the cellular immune response after immunization with the FHV-1 MLV vaccine, the cytokines levels of IFN-*γ*, IL-2, IL-4, and IL-17A in serum were detected by ELISA. Figure 3A illustrates significantly higher levels of IL-2 in the FHV-1 MLV vaccinated group than the control group (*p* < 0.01), with minimal IL-2 production in the control group after the FHV-1 challenge. However, no significant difference in IFN-*γ* and IL-17A levels was observed between the control and vaccinated groups (Figures 3B,C; *p* > 0.05). Furthermore, IL-4 levels were undetectable in all groups.

Based on the significant increase in percentages of CD8+ and CD4+ T cells and the elevated levels of IL-2 in the immunized group, it was hypothesized that the FHV-1 MLV vaccine may induces a Th1-type cell immune response in cats. To verify this, ELISpot assays detected significantly more SFCs secreting IFN-γ and IL-2, which are Th1-type cytokines, in the immunized groups compared to the control and mock groups (Figures 3D–G; *p* < 0.05). These results indicate a pronounced cellular immune response induced by the FHV-1 MLV vaccine in cats.

### 3.4 FHV-1MLV vaccine immunization significantly reduced clinical scores post-challenge

**Figure 4:**
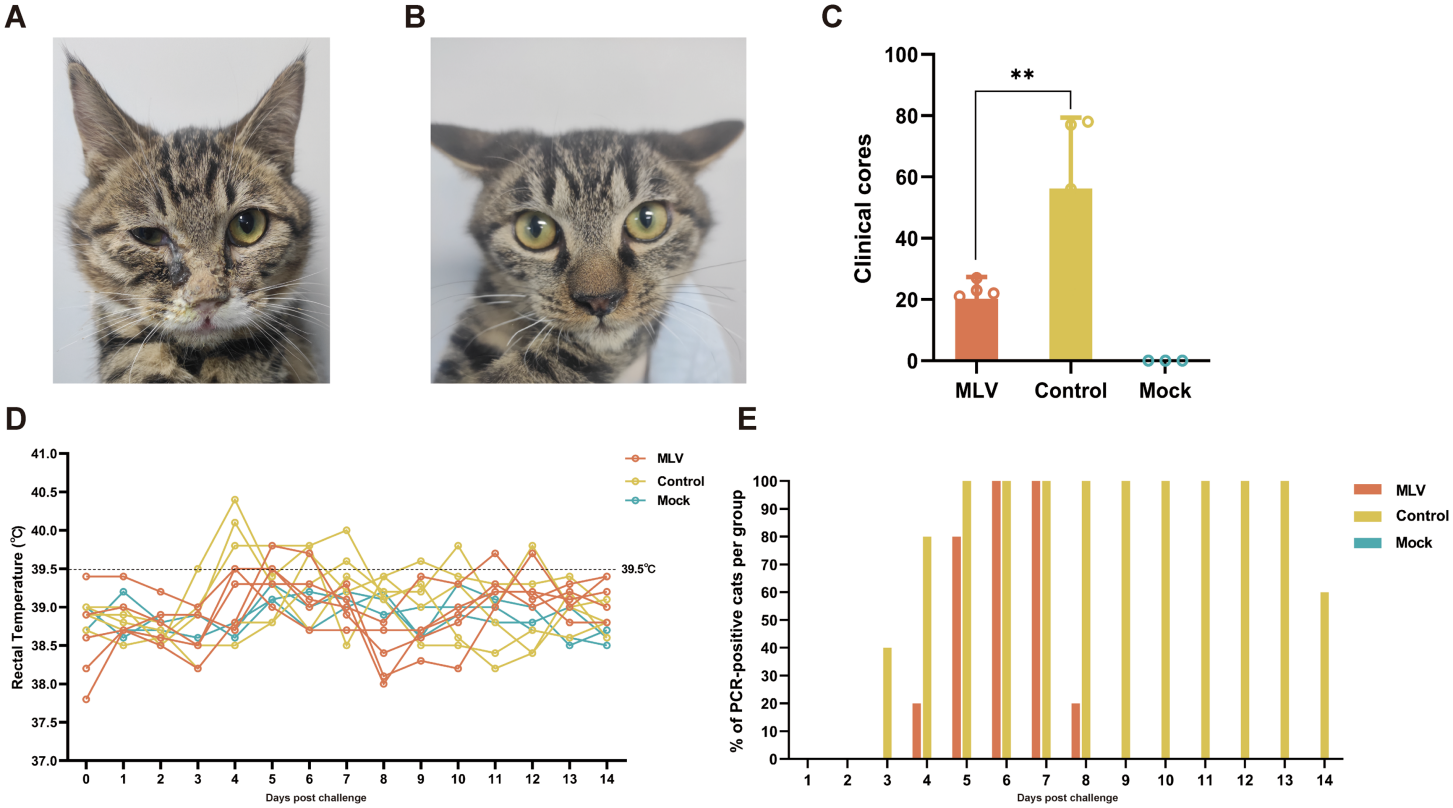
Clinical symptoms and clinical scores post FHV-1 challenge. **(A)** Cats infected with FHV-1 showed mucoid nasal or ocular discharge and blepharospasm in the control group. **(B)** Immunized cats infected with FHV-1 showed mild nasal discharge. **(C)** Statistical analysis of cumulative clinical scores in cats after FHV-1 infection, from day 36 to 49 (*n* = 13), the results were considered statistically significant at *p* < 0.05. **(D)** Rectal temperature of cats after challenge. Fever is defined as rectal temperature > 39.4°C. **(E)** The percentage of PCR-positive cats per group.

The protective efficacy of the FHV-1 MLV vaccine against FHV-1 was further evaluated by challenging cats with FHV-1 strain 64. As depicted in Figures 4A,B, cats in the control group exhibited severe clinical symptoms, including mouth breathing, mucoid ocular and nasal discharge, and sneezing. In contrast, vaccinated cats showed only mild clinical symptoms, and mock cats did not show any clinical symptoms. Clinical scores were evaluated as described previously (22, 23), and the results indicated a significant reduction in clinical scores in the vaccinated group compared to the control group (Table 1; Figure 4C; *p* < 0.05). A transient temperature increase was observed after the FHV-1 challenge in both groups, with temperatures exceeding 40°C in several control cats (Figure 4D). Moreover, as shown in Figure 4E, cats in the control group shed viral DNA in eyelid secretions for up to 2 weeks. In contrast, cats in the MLV vaccine group did not shed the virus until day 4 after infection for 5 days, and no viral shedding was detected in the mock group. These data suggest that the MLV vaccine significantly reduced clinical signs and greatly reduced FHV-1 shedding in cats.

**Table 1:** Total clinical scores after challenge with FHV-1.

Group	Animalno.	Fever	Conjunctivitis	Blepharospasm	Ocular discharge	Nasal discharge	Nasal congestion	Sneezing	Coughing	Total clinical score
MLV	1	2	0	0	0	2	3	12	2	21
	2	2	0	0	2	8	3	6	2	23
	3	0	0	0	8	6	2	6	5	27
	4	2	0	0	2	3	4	7	4	22
	5	2	0	0	0	0	0	6	0	8
Coutrol	6	3	1	1	9	25	20	11	8	78
	7	4	0	0	8	8	2	0	0	22
	8	3	0	0	7	17	11	3	7	48
	9	4	3	5	14	16	18	10	7	77
	10	3	0	0	6	18	18	7	4	56
Mock	11	0	0	0	0	0	0	0	0	0
	12	0	0	0	0	0	0	0	0	0
	13	0	0	0	0	0	0	0	0	0

**Figure 5:**
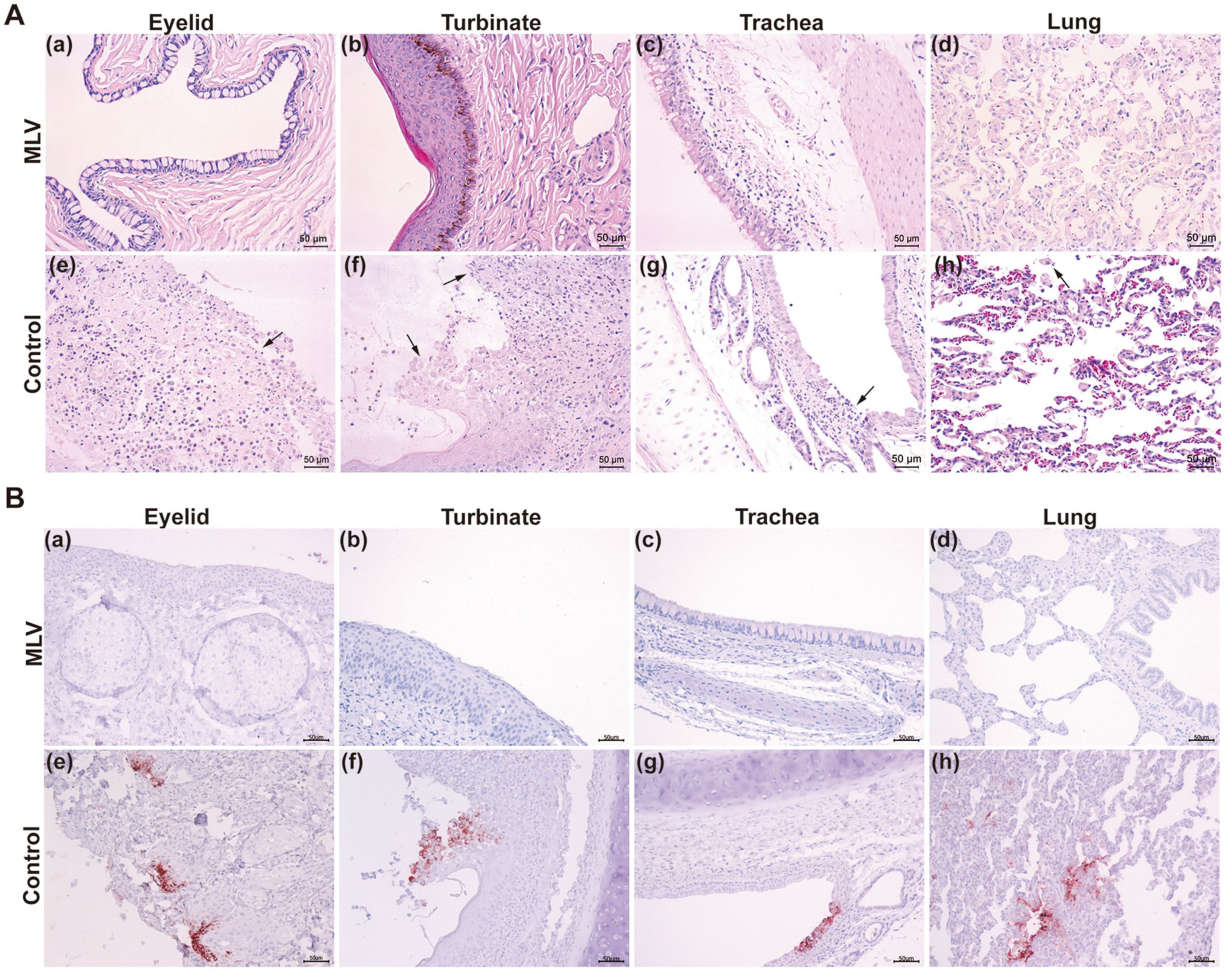
Histological changes post FHV-1 challenge. **(A)** Histopathological lesions of cats after challenge. Necropsy was performed on all cats. The eyelids, turbinates, tracheas, and lungs were collected and subjected to H&E staining. The black arrows represent tissue lesions. **(B)** IHC assays of cats after challenge.

Pathological examination 14 days post-infection revealed minimal lesions in immunized cats, while in control cats, lesions were limited to eyelids, turbinates, tracheas, and lungs, characterized by focal necrosis and desquamation of epithelial cells. Moreover, FHV-1 inclusion bodies were observed in the epithelial cells of the nasal mucosa (Figure 5A). Similarly, immunohistochemical (IHC) assays against FHV-1 showed positive staining in all control samples and negative staining in the MLV vaccine group (Figure 5B). Given that the cats in the mock group exhibited no clinical symptoms and no viral DNA was detected in their eyelid secretions, pathological examinations were not conducted on this group in consideration of animal welfare. These results indicated that the MLV vaccine confers potent protection against FHV-1 infection in cats.

### 3.5 Correlation analysis between clinical scores post-FHV-1 challenges and detection indicators

**Figure 6:**
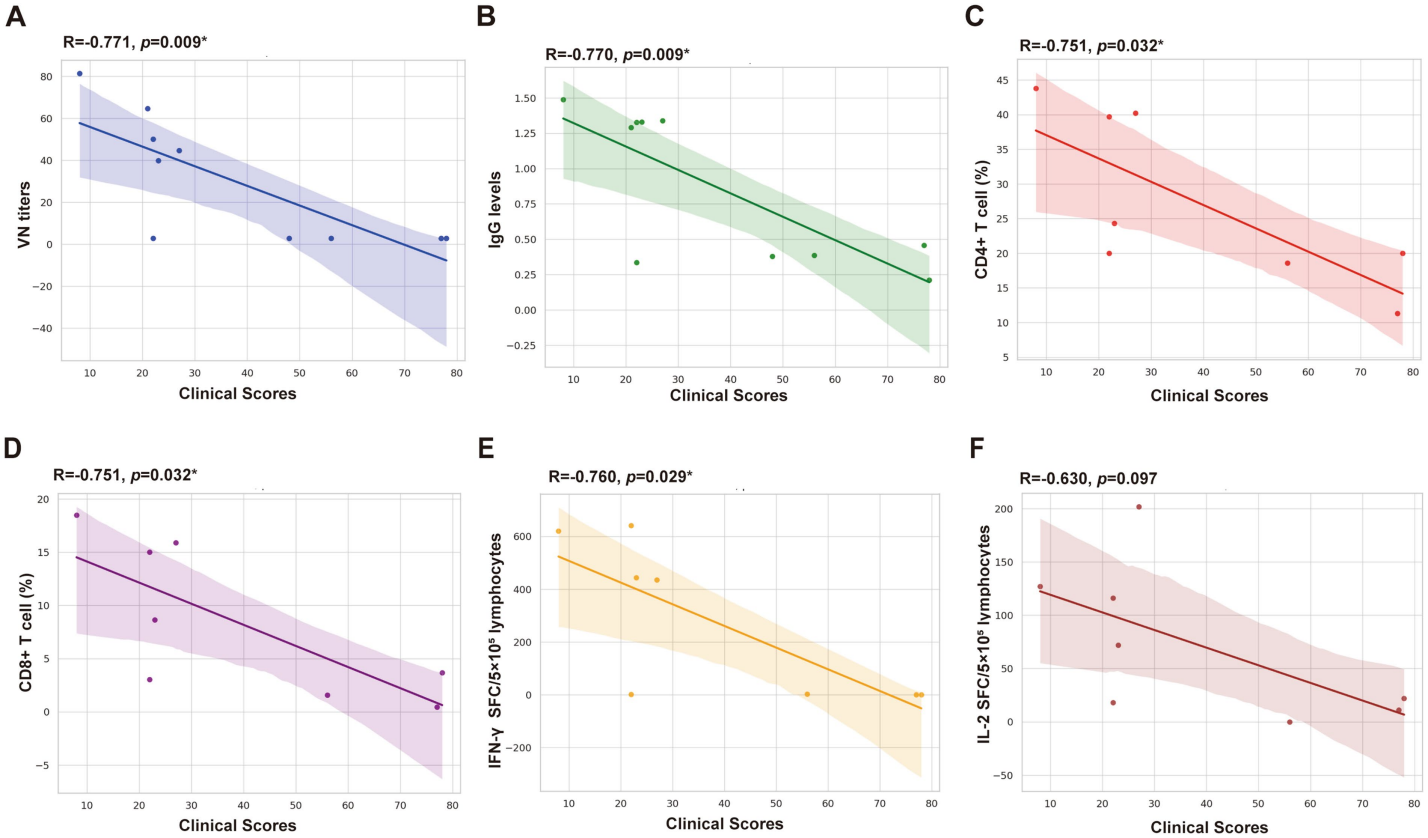
Correlation analysis between clinical scores and detection indicators. **(A)** VN titers. **(B)** IgG levels (OD 450 nm). **(C)** CD4+ T cell (%). **(D)** CD8+ T cell (%). **(E)** IFN-γ SFC/5 × 10^5^ lymphocytes. **(F)** IL-2 SFC/5 × 10^5^ lymphocytes.

Correlation analysis revealed significant negative correlations between clinical scores and VN titers (*p* < 0.05), IgG antibody titers (*p* < 0.05), IFN-*γ* SFC (*p* < 0.05), CD4 + T cells (*p* < 0.05), and CD8 + T cells (*p* < 0.05). Moderate inverse correlations were observed between clinical scores and IL-2 SFC, approaching significance (*p* > 0.05) (Figure 6).

## 4 Discussion

FHV-1 infection has become an important infectious disease that seriously endangers cats (21). Despite numerous studies on novel vaccines for FHV-1 and the availability of commercial options, the immune effects of these vaccines are inconsistent, and the disease can still develop after immunization (26). Although the FHV-1 MLV or inactivated vaccine may counteract viral infection, like FHV-1 infection, their detectable FHV-1 neutralizing antibodies after vaccination only occur in a limited number of cats with low titers (1). Several studies have confirmed this, including (27), who reported that only 22% of cats had a titer increase, with the magnitude of titer increases generally being low.

In the current study, serum neutralization antibodies and IgG levels were measured to identify the antibodies that neutralize or bind harmful particles and prevent infection. Consistent with (28), the FHV-1 MLV vaccine elicited a serological response in cats that received two doses. Typically, multiple immunizations are required to produce detectable seroconversion, regardless of whether the vaccine was attenuated or inactivated (29).

The challenge assay results align with previous research, demonstrating the vaccine’s significant clinical protection compared to the unvaccinated control group (28, 30). The vaccinated cats exhibited remarkably low clinical scores after the challenge, with symptoms limited to mild nasal or ocular discharge, mild nasal congestion, and sneezing for 2–3 days. In contrast, unvaccinated cats showed severe clinical signs. Analysis revealed a significant correlation between the clinical signs and antibodies, including VN titers and IgG levels, indicating that the humoral immune response generated by vaccination offered protection. The relationship between elevated antibody levels and reduced clinical symptoms post-FHV-1 challenge suggests that humoral immunity plays a vital role in eliminating non-bound viruses and preventing host cell invasion, thereby defending against infection (31). Consistent with other alpha herpesviruses such as equine herpesvirus-1 (EHV-1) and varicella zoster virus (VZV), it is essential to consider other immune mechanisms in preventing FHV-1 infections (32).

CD8+ T cells, or cytotoxic T lymphocytes (CTLs), are crucial in combating viral infections. Following antigen presentation, CD8+ T cells undergo clonal expansion and differentiate into effector cells, producing cytokines like IFN-*γ*. Meanwhile, CD4+ T cells differentiate into various subsets, such as Th1 cells, which produce IFN-γ and stimulate B cells to produce antibodies (33). As EHV-1 efficiently enters host cells and evades antibody neutralization in horses, T cells play a vital role in clearing replicating herpesviruses (34). CD8+ T lymphocytes primarily contribute to limiting the spread of infectious agents by recognizing and eliminating infected cells and secreting antiviral cytokines, while CD4+ T cells also play a role in this (35, 36). Consequently, the percentage of CD4+ and CD8 + T cells in the lymphocytes of immune-challenged, challenged-only, and mock cats was analyzed, which revealed a significant increase in the quantity of CD4+ T cells and CD8+ T cells in the immunized group compared to the control and mock group. These results indicate a robust immunological response induced by the FHV-1 MLV vaccine. The elevated CD8+ T cell levels suggest an effective cytotoxic response against FHV-1 infection, which is crucial for eliminating infected cells and preventing pathogen dissemination (37). The concurrent increase in both T cell populations highlights the comprehensive nature of the immune response elicited by the FHV-1 MLV vaccine, validating its effectiveness in inducing a robust cellular immune response.

Th1 T-cells (characteristically secrete IFN-*γ*, TNF-*α*, and IL-2) and CD8+ T cells contribute to the control of the alpha herpesviruses, and Th1-related cytokines like IFN-γ and IL-2 have been detected in responses to other alpha herpesviruses vaccines (24). Consistent with this, the current study depicted that the MLV vaccine induced significant levels of IL-2 in cats, with undetectable IL-4 in the serum, representing a Th1-biased cellular immune response. Although this study indicated no significant increase in IFN-*γ* levels compared to the control group, this may be attributed to the ELISA assay’s limited sensitivity, low vaccine-induced IFN-*γ* levels, or the short duration of IFN-γ in serum. To further investigate this, we employed ELISpot assay, which revealed that the MLV vaccine enhanced the secretion of IFN-γ and IL-2, associated with Th1 cells that mediate cellular immune responses and protect cells from pathogenic microorganisms (38, 39). Similarly, the live attenuated shingles vaccine Zostavax has been shown to induce a Th1-type cellular immune response that prevents VZV infection (35).

The discussed data revealed various differential factors after the immunization with the FHV-1 MLV vaccine, prompting further analysis of the correlation between clinical scores and detection indicators. The results depict a significant correlation between clinical scores and CD8+ T cells, CD4+ T cells, and IFN-*γ* SFC. These findings suggest that the MLV vaccine primarily activates the Th1-type cellular immune response, leading to CTLs differentiation and release of the IFN-γ, which clears the pathogen and confers resistance to FHV-1 infection (16, 39). The analysis of VN antibodies, IgG levels, and cytokines indicates that the FHV-1 MLV vaccine induces both humoral and cellular immune responses in cats, protecting against FHV-1 infection.

## 5 Conclusion

The findings of the study substantiate the immunological efficacy of the FHV-1 MLV vaccine. Immunized cats exhibited a Th1-type cellular immune response, as evidenced by the high levels of VN antibodies, IgG levels, and elevated levels of IFN-γ and IL-2. Furthermore, the clinical scores were substantially negatively correlated with increased IFN-γ levels that protected cats from FHV-1 infection. Overall, the FHV-1 MLV vaccine displays effective immunogenicity, and this study serves as a foundation for future FHV-1 vaccine research.

## Data Availability

The original contributions presented in the study are included in the article/supplementary material, further inquiries can be directed to the corresponding authors.
